# Predicting Acceptance of e–Mental Health Interventions in Patients With Obesity by Using an Extended Unified Theory of Acceptance Model: Cross-sectional Study

**DOI:** 10.2196/31229

**Published:** 2022-03-17

**Authors:** Vanessa Rentrop, Mirjam Damerau, Adam Schweda, Jasmin Steinbach, Lynik Chantal Schüren, Marco Niedergethmann, Eva-Maria Skoda, Martin Teufel, Alexander Bäuerle

**Affiliations:** 1 Clinic for Psychosomatic Medicine and Psychotherapy LVR-University Hospital Essen University of Duisburg-Essen Essen Germany; 2 Department of General and Visceral Surgery Alfried-Krupp Hospital Essen Essen Germany

**Keywords:** e–mental health, UTAUT, obesity, acceptance, mobile phone

## Abstract

**Background:**

The rapid increase in the number of people who are overweight and obese is a worldwide health problem. Obesity is often associated with physiological and mental health burdens. Owing to several barriers to face-to-face psychotherapy, a promising approach is to exploit recent developments and implement innovative e–mental health interventions that offer various benefits to patients with obesity and to the health care system.

**Objective:**

This study aims to assess the acceptance of e–mental health interventions in patients with obesity and explore its influencing predictors. In addition, the well-established Unified Theory of Acceptance and Use of Technology (UTAUT) model is compared with an extended UTAUT model in terms of variance explanation of acceptance.

**Methods:**

A cross-sectional web-based survey study was conducted from July 2020 to January 2021 in Germany. Eligibility requirements were adult age (≥18 years), internet access, good command of the German language, and BMI >30 kg/m^2^ (obesity). A total of 448 patients with obesity (grades I, II, and III) were recruited via specialized social media platforms. The impact of various sociodemographic, medical, and mental health characteristics was assessed. eHealth-related data and acceptance of e–mental health interventions were examined using a modified questionnaire based on the UTAUT.

**Results:**

Overall, the acceptance of e–mental health interventions in patients with obesity was moderate (mean 3.18, SD 1.11). Significant differences in the acceptance of e–mental health interventions among patients with obesity exist, depending on the grade of obesity, age, sex, occupational status, and mental health status. In an extended UTAUT regression model, acceptance was significantly predicted by the *depression score* (*Patient Health Questionnaire-8*; *β*=.07; *P*=.03), *stress owing to constant availability via mobile phone or email* (*β*=.06; *P*=.02), and *confidence in using digital media* (*β*=−0.058; *P*=.04) and by the UTAUT core predictors *performance expectancy* (*β*=.45; *P*<.001), *effort expectancy* (*β*=.22; *P*<.001), and *social influence* (*β*=.27; *P*<.001). The comparison between an extended UTAUT model (16 predictors) and the restrictive UTAUT model (*performance expectancy*, *effort expectancy*, and *social influence*) revealed a significant difference in explained variance (*F*_13,431_=2.366; *P*=.005).

**Conclusions:**

The UTAUT model has proven to be a valuable instrument to predict the acceptance of e–mental health interventions in patients with obesity. The extended UTAUT model explained a significantly high percentage of variance in acceptance (in total 73.6%). On the basis of the strong association between acceptance and future use, new interventions should focus on these UTAUT predictors to promote the establishment of effective e–mental health interventions for patients with obesity who experience mental health burdens.

## Introduction

### Background

The prevalence of people who are overweight and obese has approximately tripled since 1975, resulting in >1.9 billion adults worldwide being obese in 2016 [1]. Taking this figure and the association of obesity with serious health complications into account, obesity is considered to be a global public health crisis [2]. Obesity increases the risk of noncommunicable diseases, such as cardiovascular disease, type 2 diabetes, and even some types of cancer [3,4] and increases the chance of remaining dependent on care in nursing homes [5]. Besides several physical comorbidities, obesity is also associated with psychological distress. It elevates the risk of mental health disorders, including depression, adjustment disorders, and anxiety disorders [6-8]. For instance, previous research found that more than half of the patients with obesity experienced at least one mental disorder, resulting in low quality of life and reduced self-esteem [9,10]. Furthermore, it was shown that a high number of people with obesity have an increased risk of developing depressive disorders [11] and show symptoms of major depression [12]. In particular, discrimination against patients with obesity and weight-related stigmatization play a central role and mediate the connection to negative mental and physical health outcomes and often lead to low quality of life and poor well-being [13]. It is important to note that distress and depression inversely increase the incidence of obesity again. A very central reason for this is that individuals with obesity are often blamed for their weight and a perception among the general population is that weight stigma is justified and can motivate individuals to adopt healthy behaviors [13]. Indeed, and speaking in terms of money, obesity causes large economic burdens for health care systems [14].

However, effective interventions for obesity management are scarce because they are often impractical to implement in the health care system [15]. The social stigma that accompanies obesity and the impediments in mobility of older patients with obesity are only 2 of many barriers preventing good professional patient care [15]. In particular, long-term psychotherapeutic and psychosocial care of people with obesity are often not possible owing to limited health care opportunities. As a result, the development and implementation of cost-effective and low-threshold approaches to handle elevated psychological distress and existing mental health disorders are essential.

E–mental health interventions are an effective and innovative approach that can circumvent the aforementioned barriers hindering the health care system. Especially during the COVID-19 pandemic, the need for contact-free, low-threshold, and easily accessible approaches in health care has become increasingly evident [16]. During this crisis, many institutions in the health care system tried to develop possibilities to serve their patients via videoconferencing or other digital methods [17,18]. Innovative e–mental health interventions offer a low-threshold, time- and location-flexible, and often anonymous alternative to traditional face-to-face therapy. Although the implementation of e–mental health interventions in Germany is in its early stage [19], multiple studies have shown effects comparable with that of face-to-face therapy [19,20]. Existing e–mental health interventions for patients with a variety of mental disorders mostly include psychoeducational interventions and web-based tasks designed for cognitive behavioral, psychodynamic, or acceptance and commitment therapy [21]. Acceptance of participants who participated in such interventions and their satisfaction with such interventions has often been considerable [21,22]. A recent study revealed that patients who had undergone bariatric surgery were very positive about eHealth interventions in their follow-up care [23]. Particularly after bariatric surgery, eHealth interventions can be effective in postoperative weight maintenance and in the reduction of eating disorder symptoms [24]. Although the proven user acceptance for e–mental health interventions [25] and the reasonable assumption that patients with obesity affected by mental health disorders would benefit from easily accessible and effective e–mental health interventions to support their mental well-being, the current opportunities are still in their infancy. Therefore, it is important to explore the patient-specific needs because newly developed interventions need to be tailored to these needs to foster user acceptance and treatment adherence [26].

Besides the advantages and resources described above, there are barriers to using eHealth interventions that should not be neglected, which will now be examined in more detail. In addition, previous research has shown that various predictors seem to influence patient acceptance of e–mental health interventions. A cross-sectional survey in 2016 demonstrated that the acceptance of web-based aftercare among inpatients (groups mentioned below) is low [21]. The highlighted factors that were able to significantly predict acceptance were social influence (SI), performance expectancy (PE), and effort expectancy (EE; 3 predictors of the Unified Theory of Acceptance and Use of Technology [UTAUT] model, which will be described in more detail in the following sections). Although high acceptance correlated with (young) age, (high) education, (high) level of information, and experience, the stress caused by permanent availability was associated with low acceptance; however, the effect was very small. Other factors influencing the acceptance of web-based aftercare were different patient groups (psychosomatic, cardiologic, orthopedic, pediatric, and substance-related disorders) [21] and current employment status [27].

**Figure 1:**
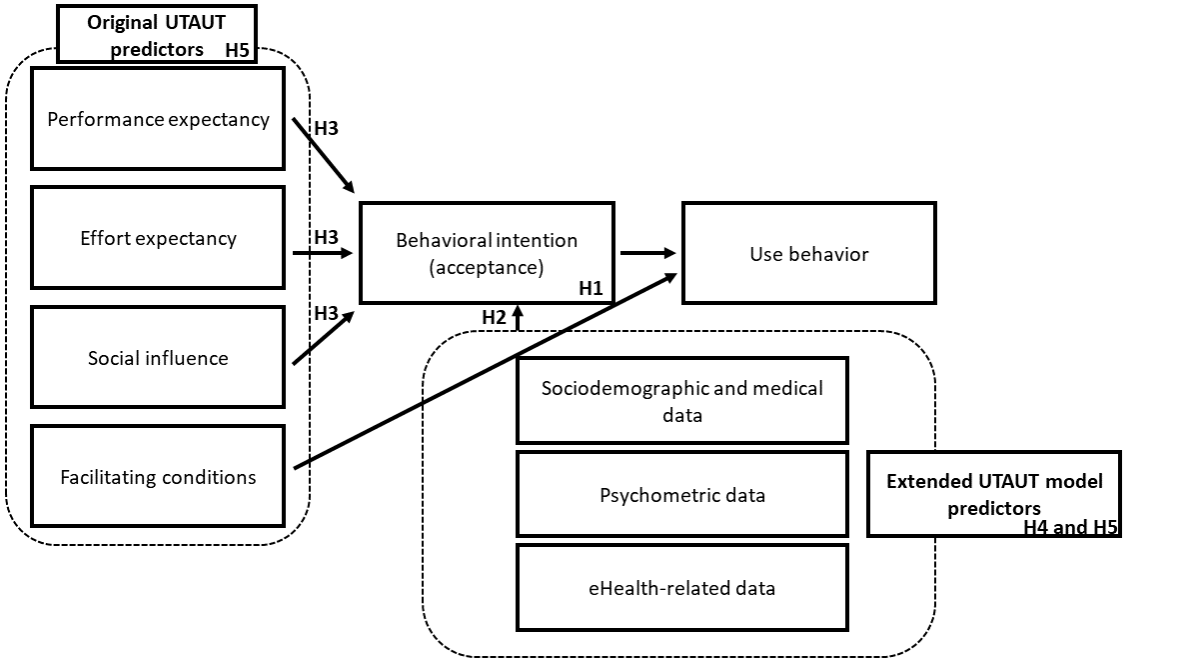
Unified Theory of Acceptance and Use of Technology (UTAUT) model and the extended model predictors including hypotheses (H) 1-5.

In addition, 2 research studies focusing on the patient’s perspective explored the following barriers of e–mental health interventions reported by health care providers: the lack of guidance through therapeutic relationship, limitation of communication, and control or concerns about data security [28,29]. To minimize the supposed disadvantages of e–mental health interventions for patients and health care professionals and to focus on possible advantages, specific research in this area is necessary. As the implementation and uptake of e–mental health interventions are still low, the acceptance of such interventions and the barriers hindering them make their use less likely and need to be further evaluated [30]. Most studies assessing the acceptance of eHealth interventions are not based on valid measurement constructs [31,32]; therefore, for this study, it is important to determine that the results are evaluated based on solid operationalization and theoretical ramifications. Therefore, the UTAUT is used in this study [33], as it has already been used in research on eHealth to validly identify the determinants of acceptance [33]. The UTAUT model consists of four main predictors: *PE, EE, SI, and facilitating conditions (FCs)* [34]. It can be used to assess acceptance of an eHealth intervention or other technological systems using the first 3 core predictors, whereas acceptance itself is operationalized as *behavioral intention (BI)* to use such interventions on a fifth scale. PE describes the degree to which an individual believes that he or she will benefit from using the intervention. EE is defined as the degree of ease associated with the use of the technology. A person’s assessment of the extent to which their relevant social contacts (ie, family and friends) would approve the intervention is indicated by SI. FC is defined as the degree to which an individual believes that an organizational and technical infrastructure exists to support the use of the system [33]. The acceptance (intention to use; Figure 1) is predicted by the three main predictors (*PE, EE, and SI*), whereas actual use behavior is predicted by the intention to use and FCs. Researchers have pointed out that the UTAUT model needs to be explored and validated in different target groups [21]. A study using the UTAUT model for acceptance measurement conducted by Hennemann, Beutel, and Zwerenz in 2017 [30] revealed that acceptance of e–mental health interventions was quite low and acceptance of web-based aftercare was moderate and did not differ between age groups. Significant predictors of high acceptance are PE, SI, and treatment-related internet and mobile use [30]. A further study in 2019 found that FCs and perceived usefulness were associated with increased eHealth activity, whereas SI was not associated with eHealth use [35]. In 2015, de Veer et al [36] found that 63.1% of elderly people (aged between 57 and 77 years) included in their study would use an eHealth app (moderate acceptance) by using the UTAUT model. In this case, the model showed that PE, EE, and self-efficacy were highly related to acceptance of eHealth intervention, whereas SI was not [36]. Although previous research has focused on the acceptance of e–mental health interventions, it has rarely measured acceptance using validated constructs, making it important to use the UTAUT model in future research. In addition, because of the various findings to date on the different variables that might predict acceptance, more research with the UTAUT model is necessary. Research studies are needed, on the one hand, to establish relationships between the individual components, the use behavior, and the acceptance of e–mental health interventions in patients with obesity and, on the other hand, to validate the UTAUT model in other patient cohorts than the ones studied so far.

### Objectives

This study aims to assess the acceptance of e–mental health interventions in patients with obesity (grades I, II, and III) and to explore the underlying, influencing factors determining the acceptance. We use the established UTAUT model and extend it to accomplish the abovementioned goal. Previous studies have already shown that acceptance is associated with sociodemographic variables such as age and sex [21,37]. In addition, there were differences in acceptance depending on the mental health status of the patients [37,38] and evidence that patients who have already undergone bariatric surgery show high acceptance of eHealth interventions [21]. Previous research has not yet addressed patients with obesity and their acceptance of e–mental health interventions. To identify additional variables associated with acceptance of e–mental health interventions, this study includes several obesity-specific factors (eg, bariatric surgery and the grade of obesity). Previous research has examined acceptance and various predictors in other patient groups, leading us to propose the following assumptions for our study:

- Hypothesis 1: In accordance with previous research in other patient groups [21,30,38], it is assumed that the overall acceptance of e–mental health interventions in patients with obesity is moderate.
- Hypothesis 2: Moreover, we assume that we will find group differences in the acceptance of e–mental health interventions depending on sex, age, grade of obesity, occupational status, mental disorder, outpatient psychotherapy, and previously performed bariatric surgery [21,25,27].
- Hypothesis 3: We postulate a positive relation between the UTAUT factors (SI, PE, and EE) and the acceptance of e–mental health interventions for people with obesity [21,33,35,36] (Figure 1).
- Hypothesis 4: Furthermore, we assume that, in addition to sociodemographic and medical factors, psychometric data and eHealth-related data, for example, internet anxiety (negative) and experience with e–mental health interventions (positive), significantly explain variance in the acceptance of e–mental health interventions among patients with obesity [21].
- Hypothesis 5: In a comparison between the restrictive UTAUT model and our extended UTAUT model, we hypothesize a significant difference in the explanation of variance.

The results of this study could significantly accelerate the process of implementing and adapting e–mental health interventions for specific patient groups, such as patients with obesity. Especially considering how the current pandemic has caused additional mental health burdens, more efficient and easily available ways to provide psychological support should be developed.

## Methods

### Study Design and Participants

A cross-sectional approach was implemented to measure the acceptance of e–mental health interventions and its underlying predictors in a sample of patients with obesity based on the UTAUT.

Participants were recruited from July 2020 to January 2021 at the Obesity Center of Alfried Krupp Hospital and via social media platform groups such as Facebook, exclusively directed toward patients who are seeking, undergoing, or have already undergone bariatric surgery and were aged ≥18 years. Other eligibility requirements were good command of the German language, internet access, and BMI >30 kg/m^2^ (diagnosis of obesity). The classification of obesity grades according to the World Health Organization is as follows: (1) obesity grade I (BMI 30-34.9 kg/m^2^), (2) obesity grade II (BMI 35-39.9 kg/m^2^), and (3) obesity grade III (BMI ≥40 kg/m^2^) [39]. The processing time of the web-based survey, consisting of 68 items in total, was approximately 18 minutes. No financial compensation was offered. Electronic informed consent was obtained before the survey began, and participation was completely anonymous and voluntary. Of 996 participants who started the survey, 643 (64.6%) participants completed it. A total of 30.3% (195/643) of participants were underweight, normal weight, or overweight but not with BMI >30 kg/m^2^; thus, they were excluded from this study. This resulted in a total sample of 69.7% (448/643) of participants, with no one being excluded owing to additional criteria. 

### Ethics Approval

The survey was conducted in accordance with the Declaration of Helsinki, and the Ethics Committee of the Essen Medical Faculty (19-89-47-BO) agreed to conduct the study.

### Measures

#### Overview

The survey contained items of sociodemographic, medical, and mental health data. In addition, we used a modified UTAUT questionnaire (based on previous adaptations) to assess the acceptance of e–mental health interventions and the resources of and barriers to eHealth use. The exact questionnaire is presented in Textbox 1. To assess the mental health of the participants, we used validated instruments such as the Eating Disorder Inventory-2–Bulimia (EDI-2–B), Eating Disorder Examination-Questionnaire 8 (EDE-Q8), and Patient Health Questionnaire-8 (PHQ-8).

**Scales and adapted items of Unified Theory of Acceptance and Use of Technology and references of original studies (italicized verbalizations have been adapted).**

**Behavioral intention (acceptance)**

- “I would like to try a psychological online intervention.” [26,40]
- “I would use a psychological online intervention if offered to me.” [26,40]
- “I would recommend a psychological online intervention to my friends.” [38]

**Social influence**

- “People close to me would approve the use of a psychological online intervention.” [26,34,40]
- “My general practitioner would approve of a psychological online intervention.” [26,40]
- “My friends would approve of a psychological online intervention.” [21]

**Performance expectancy**

- “A psychological online intervention could improve my general well-being.” [26,40]
- “A psychological online intervention could help me with stress.” [26,40]
- “A psychological online intervention could help me improve my personal (psychological) health.” [26,40]

**Effort expectancy**

- “The use of a psychological online intervention would not be an additional burden to me.” (self-constructed)
- “A psychological online intervention would be easy to operate and comprehend.” [26,33,40,41]
- “I could arrange using a
  psychological online intervention
  in my everyday life.” [21]

#### Sociodemographic and Medical Data

Sociodemographic and medical data were assessed using items on age, sex, marital status, having children, occupational status, educational level, physical illness, mental disorder, and medication. In addition, there were items on data related to obesity and its management (weight, height, BMI, grade of obesity, comorbidities, and bariatric surgery).

#### Acceptance, eHealth Use, and UTAUT Predictors

To assess the acceptance of e–mental health interventions and its underlying factors, a modified version of the UTAUT model (Textbox 1) and several items for the measurement of internet use, internet anxiety, and attitudes toward and experiences with web-based interventions were used. The UTAUT questionnaire consists of 12 items and answers are given on a 5-point Likert scale (ranging from 1=totally disagree to 5=totally agree). Three items measure the underlying predictors of acceptance and acceptance itself, which are operationalized as intention to use (BI). Cronbach α values in this study were .88 for acceptance (BI), .84 for SI, .93 for PE, and .82 for EE, proving high internal consistency.

To assess the eHealth use of the participants, items such as the duration of use of media such their smartphone or tablet and previous experiences with eHealth interventions were asked. To record how confident the participants felt in using digital media, they were asked to rate their confidence on a scale of 1 (very unsafe) to 5 (very safe). The perceived stress caused by permanent availability via mobile phone or email was surveyed on a scale of 1 (strongly disagree) to 5 (strongly agree). The participants’ internet anxiety was assessed using a set of 3 items (already used in previous studies), of which a mean value was calculated on a scale from 1 to 5, with 5 indicating very high internet anxiety. Cronbach α for this instrument in this study was .76, which indicates a sufficient internal consistency.

#### Assessment Using EDI-2–B

The EDI-2–B consists of 7 items assessing symptoms of bulimia (especially binge eating) on a 6-point Likert scale (1=never to 6=always) [42]. The sum score has a minimum of 7 points and a maximum of 42 points. Cronbach α in this study was .81, indicating high internal consistency.

#### Assessment Using EDE-Q8

The EDE-Q8 is a short version of the EDE-Q and comprises four subscales: restraint, eating concern, shape concern, and weight concern [43]. In this abbreviated version, 2 items refer to each scale (8 items in total), thus ensuring optimal internal consistency, one-dimensionality, and even coverage of the EDE-Q subscales. It consists of 5 items assessing eating disorder psychopathology in the past 28 days on a 7-point Likert scale (ranging from 0=not any day to 6=every day) and 3 items assessing the occurrence and frequency of core eating disorder behavior on a scale (from 0=never to 6=every time). Cronbach α in this study was .78, which indicates a sufficient internal consistency.

#### Assessment Using PHQ-8

The PHQ-8 measures depression symptoms via 8 items on a 4-point Likert scale (0=not at all to 3=nearly every day) [44]. A score ≥10 indicates major depression symptoms. Cronbach α in this study was .85, indicating high internal consistency.

### Statistical Analyses

Data analysis was performed using SPSS Statistics 26 software (IBM). First, the sum scores and mean scores for scales PHQ-8, EDE-Q-8, and EDI-2–B were computed. Second, the internal consistencies for the different psychometric questionnaires were calculated and descriptive statistics were performed. Third, the acceptance was computed (mean value) and its distribution was assessed.

Acceptance (BI) of the UTAUT model (scale 1-5) was categorized by mean as low (1-2.34), moderate (2.35-3.67), and high (3.68-5) acceptance. Percentage and absolute count in categories were calculated. To highlight group differences in age, four age categories were formed before analysis: (1) 18-34 years, (2) 35-44 years, (3) 45-54 years, and (4) 55-69 years. The BMI of the participants was calculated by dividing their body weight by their height in meters squared. The means of acceptance (BI) were compared between groups regarding sociodemographic and medical data with 2-tailed *t* tests and analyses of variance (ANOVAs) to also include variables with multiple categories. The normal distribution of acceptance was examined using the Kolmogorov-Smirnov test, skewness, and kurtosis and graphically via a histogram including a normal distribution curve. All measures detected violations against normal distribution. However, we still used parametric tests for various reasons. According to the central limit theorem, the sampling distribution of the mean of a variable can be safely assumed to be normal if the variable and its mean are normally distributed in the population and the sample size is sufficiently large. We consider our sample size of 448 as sufficient because some studies suggest that such an effect already emerges at the sample size of 30 [45]. Moreover, other researchers found that acceptance distributions in general did not differ from normal distribution, which indicates that the variable acceptance might be normally distributed in the population [38]. In addition, 2-tailed *t* tests and ANOVAs are considered to be robust against violations, assuming normal distribution [46].

Using multiple hierarchical regression, the predictive model of acceptance was tested by using the enter method. The following predictors were included blockwise: (1) sociodemographic and medical data, (2) psychometric data, (3) eHealth-related data, and (4) UTAUT predictors (Figure 1). In addition, the full model was tested against the restricted UTAUT model with the UTAUT predictors (*PE, EE,* and *SI*) only. No multicollinearity could be detected because all the variance inflation factor values for testing multicollinearity were <5. The QQ plots of the residuals were visually inspected and showed no signs of violations against normality; therefore, normal distribution of the residuals can be assumed. Homoscedasticity was proven based on a scatter plot of the standardized residuals and adjusted predicted values.

For every ANOVA and 2-tailed *t* test, the level of significance was set at .05. In addition, post hoc tests were used to describe differences between the groups.

## Results

### Sociodemographic, Medical, and Psychometric Data

A total of 448 individuals participated in this study, of which 403 (89.9%) were women and 45 (10%) were men. The mean age was 44.69 years and age ranged from 18 to 69 years. Marital status for most participants was married (246/448, 54.9%) or living in a partnership (77/448, 17.2%). Of the 448 participants, 67 (14.9%) participants reported being single. Of the 448 participants, 98 (21.9%) participants had graduated from high school, 55 (12.3%) had graduated from college, and 210 (46.9%) had graduated from junior high school. In all, 66.3% (297/448) of the participants were employed at the time of participation and 33.7% (151/448) of the participants were unemployed.

A mental disorder was reported by 37.5% (168/448) of the participants. The diagnosis of depression was mentioned most frequently (58/168, 34.5%). There were 20.5% (92/448) of participants in outpatient psychotherapy owing to a mental disorder. In all, 22.8% (102/448) of the participants reported currently taking a psychiatric medication. The presence of a physical disease (other than obesity) was reported by 64.9% (291/448) of the participants. Frequently mentioned diseases (other than obesity) were diabetes, hypertension, and joint pain (182/448, 40.6%). Of the 448 participants with obesity, a total of 82 (18.3%) participants had obesity grade I, 88 (19.6%) participants had obesity grade II, and 278 (62.1%) participants had obesity grade III. Totally, 44.6% (200/448) of the participants had already undergone bariatric surgery. Of these 200 participants, 80 (40%) participants were part of the grade III obesity group. Of the 448 participants, 170 (37.9%) participants indicated that they planned to have bariatric surgery. Only 17.4% (78/448) participants neither had surgery nor planned to do so. Of the 200 participants who had already undergone surgery, 107 (53.5%) had opted for a gastric tube.

Of the 448 participants, 31 (6.9%) participants reported using the internet for private use for 0-1 hour per day, 94 (20.9%) participants used the internet for 1-2 hours per day, 119 (26.6%) participants reported a use time of 2-3 hours per day, 88 (19.6%) participants reported a use time of 3-4 hours per day, and approximately 116 (25.9%) people used the internet for >4 hours per day for private needs. The mean score for confidence in using digital media in this study was high (mean 4.08, SD 0.97). The stress experienced by the participants owing to permanent availability via mobile phone or email was moderate (mean 2.59, SD 1.24). Internet anxiety among participants in the study was low (mean 1.60, SD 0.76). Experience with e–mental health interventions was reported by 19.6% (88/448) of participants, whereas the remaining 80.4% (360/448) of the participants had no experience with such interventions.

The sum score of the EDI-2–B scale (mean 15.62, SD 0.87) was high compared with the norm values defined by Thiel and Paul [42], with a sample of 40.8% (183/448) of the participants (mean 11.59, SD 4.00) [43]. The sum score of EDE-Q8 (mean 3.89, SD 1.22) was also relatively high compared with healthy control patients from previous study by Hilbert et al [43] with a sample of 91.3% (409/448; mean 1.44, SD 1.22) and indicated eating disorder symptoms [44]. Using the recommended cutoff in the literature of ≥10, analyses of PHQ-8 (mean 10.10, SD 5.64) showed, that 49.6% (222/448) of the participants in this study might experience symptoms of major depression. In a very large random comparison sample (N=198,678) from a study in 2009, 8.6% of the participants experienced depressive symptoms (PHQ-8 sum score >10) [44].

### Hypotheses 1 and 2: Acceptance as a Function of Sociodemographic and Medical Data

The general acceptance of e–mental health interventions was moderate (mean 3.18, SD 1.11). We divided the sample of 448 participants into groups by their degree of acceptance, with the results being as follows: 116 (25.9%) participants showed low acceptance, 199 (44.4%) showed moderate acceptance, and 133 (29.7%) showed high acceptance.

Table 1 contains the acceptance scores as a function of sociodemographic and medical data. Acceptance differed significantly between sexes (*t*_446_=2.35; *P*=.02), with higher acceptance among women than among men. In addition, there were significant differences in acceptance by the different age groups (*F*_3,444_=2.75; *P=*.04), with the highest acceptance in the middle-age group (35-44 years) and the lowest acceptance in the oldest group (55-69 years). However, the post hoc test of ANOVA showed that only age group II (35-44 years) and age group IV (55-69 years) differed significantly (*P=*.03). There was a significant difference in acceptance among the 3 obesity groups, with high acceptance level among people with obesity grade II (*F*_2,445_=6.59; *P=*.002). The post hoc test of ANOVA revealed that the obesity grade I group was significantly different from grades II (*P*=.004) and III (*P*=.003). There was no significant difference in acceptance between grade II and grade III (*P*=.99). The occupational status (employed) was also significantly associated with higher acceptance ratings (*t*_446_=2.40; *P=*.02) compared with those participants who were currently unemployed. In addition, participants with a mental disorder displayed significantly higher acceptance than those without a mental disorder (*t*_446_=2.02; *P=*.02). There were no significant differences in acceptance regarding the variables such as bariatric surgery and outpatient psychotherapy.

**Table 1:** Differences in acceptance by sociodemographic and medical data (N=448).

Variable			Values, n (%)		Values, mean (SD)		*t* test (*df*)		*F* test (*df*)		*P* value
**Sex**							2.35 (446)		N/A^a^		.02
	Women	403 (89.9)		3.23 (1.09)							
	Men	45 (10)		2.78 (1.24)							
**Age (years)**							N/A		2.75 (3,444)		.04
	18-34	86 (19.1)		3.15 (1.12)							
	35-44	131 (29.2)		3.39 (1.12)							
	45-54	140 (31.3)		3.16 (1.14)							
	55-69	91 (20.3)		2.96 (1.03)							
**Grade of obesity**							N/A		6.59 (2,445)		.002
	I	82 (18.3)		2.79 (1.06)							
	II	88 (19.6)		3.34 (1.06)							
	III	278 (62.1)		3.25 (1.12)							
**Occupational status**							2.40 (446)		N/A		.02
	Employed	297 (66.3)		3.28 (1.11)							
	Unemployed	151 (33.7)		3.01 (1.11)							
**Mental disorder**							2.02 (446)		N/A		.02
	Yes	168 (37.5)		3.35 (1.05)							
	No	280 (62.5)		3.09 (1.14)							
**Bariatric surgery**							N/A		1.04 (2,445)		.35
	Yes, executed	200 (44.6)		3.11 (1.10)							
	Already planned	170 (37.9)		3.28 (1.16)							
	No	78 (17.4)		3.19 (1.07)							
**Outpatient psychotherapy**							0.795 (446)		N/A		.78
	Yes	92 (20.5)		3.21 (1.16)							
	No	356 (79.5)		3.11 (1.16)							

^a^N/A: not applicable.

### Hypotheses 3 and 4: Predictors of Acceptance

The multiple hierarchical regression analysis revealed that the sociodemographic and medical predictors included in the first step explained 7.1% (*R*²=0.071; *F*_5,442_=6.74; *P*<.001) of the variance in acceptance. Thereby, sex (*β*=−0.13; *P=*.006), BMI (*β*=.13; *P*=.007), presence of a mental disorder (*β*=.16; *P*<.001), and occupational status (*β*=−0.18; *P*<.001) significantly predicted acceptance. The psychometric predictors included in the second step (*R*²=0.127; *F*_8,439_=7.98; *P*<.001) increased the explained variance significantly (Δ*R*²=0.056; *F*_3,439_=9.431; *P*<.001), whereby the depression sum score (PHQ-8; *β*=.12; *P=*.04) and the EDE-Q8 score (*β*=.13; *P=*.02) significantly predicted acceptance. In the third step, when entering the eHealth-related predictors, the explained variance in acceptance increased significantly to 15.4% (*R*²=0.154; *F*_13,434_=6.094; *P*<.001), but only the stress owing to constant availability on the mobile phone or via email was a significant predictor in this step (*β*=.12; *P=*.02). The UTAUT predictors included in the last step (*R*²=0.736; *F*_16,431_=75.26; *P*<.001) changed the explained variance significantly by 58.2% (Δ*R*²=0.582; *F*_3,431_=317.28; *P*<.001), resulting in a total percentage of 73.6% for the explained variance. *PE* (*β*=.45; *P*<.001), *EE* (*β*=.22; *P*<.001), and *SI* (*β*=.27; *P*<.001) significantly predicted acceptance. In the overall model (step 4), in addition to the 3 UTAUT predictors, 3 additional variables from the extended model can be established as significant predictors of the acceptance of e–mental health interventions in people with obesity: *depression sum score* (PHQ-8; *β*=.07; *P*=.03), *stress owing to constant availability via mobile phone or* email (*β*=.06; *P*=.02), and *confidence in using digital media* (*β*=−0.06; *P*=.04). Table 2 presents the regression parameters of the hierarchical regression model of acceptance. Multimedia Appendix 1 illustrates the full model of the hierarchical regression.

**Table 2:** Hierarchical regression model of acceptance (N=448).

Predictor^a^			*β* ^b^		B^c^		T		*R*²^d^			Δ*R*^²e^			*P* value	
**Step 1: sociodemographic and medical variables**										0.071			0.071			
	Sex	−0.130		−0.480		−2.788								.006		
	Age	−0.058		−0.006		−1.257								.21		
	Mental disorder	.156		.179		3.235								.001		
	BMI	.126		.017		2.760								.007		
	Occupational status	−0.182		−0.430		−3.760								<.001		
**Step 2: psychometric variables**										0.127			0.056			
	EDI-2–B^f^	.072		.013		1.384								.17		
	EDE-Q8^g^	.126		.115		2.293								.02		
	PHQ-8^h^	.124		.025		2.080								.04		
**Step 3: eHealth-related variables**										0.154			0.027			
	Stress owing to permanent availability	.112		.100		2.285								.02		
	Internet anxiety	−0.045		−0.067		−.884								.38		
	Information about eHealth interventions	.083		.088		1.664								.10		
	Experience with eHealth interventions	.092		.257		1.854								.06		
	Confidence in using digital media	.069		.079		1.368								.17		
**Step 4: UTAUT^i^ predictors**										0.736			0.582			
	Performance expectancy	.454		.484		11.683								<.001		
	Effort expectancy	.219		.268		5.913								<.001		
	Social influence	.271		.361		7.956								<.001		

^a^In steps 2, 3, and 4, only the newly included variables are presented.

^b^Standardized coefficient *β*.

^c^Unstandardized coefficient *β*.

^d^Determination coefficient.

^e^Changes in *R*².

^f^EDI-2–B: Eating Disorder Inventory-2–Bulimia.

^g^EDE-Q8: Eating Disorder Examination-Questionnaire 8.

^h^PHQ-8: Patient Health Questionnaire-8.

^i^UTAUT: Unified Theory of Acceptance and Use of Technology.

### Hypothesis 5: UTAUT Versus Extended UTAUT Model

For hypothesis 5, we aimed to determine whether our full model (*R^2^*=0.736) is better in explaining the variance in acceptance of e–mental health interventions than a restricted model (*R^2^*=0.718), including only the UTAUT predictors of acceptance (*PE*, *EE,* and *SI*). Comparison of both models revealed a significant difference in the explained variance (*F*_13,431_=2.366; *P*=.005), which means that the extended UTAUT model provides high variance explanation in the acceptance of e–mental health interventions owing to the additional included variables.

## Discussion

### Principal Findings

This study assessed the acceptance of e–mental health interventions among patients with obesity and explored the factors influencing acceptance. First, it is important to note that all hypotheses (1-5) were confirmed in this study. The overall acceptance of e–mental health interventions among people with obesity was moderate, with 29.7% (133/448) of participants indicating high acceptance, 44.4% (199/448) indicating moderate acceptance, and only 25.9% (116/448) indicating low acceptance of e–mental health interventions. Regarding the second hypothesis, the data yielded evidence for small but significant differences in acceptance depending on the obesity grade, with the highest acceptance in the grade II obesity group. Participants with obesity grade I differed significantly from those with obesity grades II and III in terms of their acceptance of e–mental health interventions, with patients with obesity grade I exhibiting significantly low acceptance. Employed participants had a significantly higher acceptance of e–mental health interventions than unemployed participants, and participants with a mental disorder also showed significantly high acceptance; however, these differences were mostly slight. Regarding the third and fourth hypotheses, the following can be reported. In the extended regression model, the acceptance of e–mental health interventions was significantly predicted by depressive symptoms (PHQ-8). In addition, the stress caused by permanent availability via mobile phone or email was found to be a significant predictor of acceptance. Moreover, in the second step of the hierarchical regression, we used psychometric data that already explained a small part of the variance. This was different in previous research, which neglected the inclusion of psychometric data and only included eHealth-related data [21]. In addition to the 3 UTAUT predictors, confidence in using digital media was identified as a significant predictor in the regression model. Regarding the fifth hypothesis, the evidence is that the UTAUT predictors (restricted model with UTAUT predictors only) reached a high level in explained variance, whereas the extended UTAUT model (16 predictors) clarified slightly but with significantly more variance in patients with obesity (Table 2).

### Comparison With Previous Work

Although previous studies have highlighted the mental health burden among patients with obesity, research regarding the acceptance and use of specific e–mental health interventions, especially with validated measures (eg, UTAUT), is still very scarce for the examined patient group [19]. The general acceptance of e–mental health interventions shown by participants was higher in this study than in previous studies [21,47,48], which also did not exclusively survey patients with obesity, who are a special patient group owing to their psychological and physical problems. We conducted a well-powered study comparable with previous research efforts, which often had a small sample, lacked valid measurement instruments, and captured very few variables that could be important for the acceptance of e–mental health interventions. We aimed to rectify these shortcomings, especially by using the validated measurement of acceptance of e–mental health interventions using the UTAUT model; adding sociodemographic, psychometric, and eHealth-related variables; and recruiting a substantial sample.

Studies that have previously examined the acceptance of e–mental health interventions in general or in specific patient groups have been able to identify the following variables to be significantly associated with acceptance: in addition to age [21,37,48], there is evidence of sex [37,48], anxiety [38], internet anxiety [38], experience with e–mental health interventions [21,37,47], education [21,37,48], experiencing a mental illness [37], and duration of type 2 diabetes [48]. Similar to previous research, the acceptance ratings in this study were significantly associated with sex. However, contrary to previous research by Hennemann et al [21] and Roelofsen et al [49], the acceptance ratings in this study were significantly higher for women than for men. We attribute this finding particularly to the topic of losing weight, dealing with their own bodies, and the psychological factors in this regard, which more often plays a major role in the lives of women [50]. Age was also significantly associated with acceptance [21,49], whereby the middle-age group (35-44 years) differed significantly from the oldest group (55-69 years) in their acceptance, which could be because older people are less familiar with the internet and digital media. In addition, this study was successful in detecting the associations of the grade of obesity and experiencing a mental disorder with the acceptance of e–mental health interventions. According to previous research, it can be assumed that patients with a high BMI are more psychologically burdened; thus, it can be hypothesized that patient’s acceptance of digital interventions also increases with higher weight (eg, owing to the immobility of the patient group), as these patients are more likely to be searching for psychological interventions and experiencing high levels of distress [51-53]. In this study, the level of distress and associated openness to psychological interventions among people with mental disorders also could possibly lead people who are currently experiencing a mental disorder to report high acceptance of e–mental health interventions. This could be in part because people with mental disorders are directly affected by the lack of psychosocial treatment possibilities and are more likely to be grateful to receive any low-threshold interventions to improve their symptoms. A practical implication that arises from this is the tailoring of specific eHealth interventions, especially to the psychological distress of these patients. Previous studies have used the UTAUT model to identify predictors of acceptance and use of internet-based interventions so that the following significant predictors could be identified: *PE* [21,48,54,55], *EE* [21,48,54,55], and *SI* [21,54,55].

The fourth core predictor of UTAUT has been named as *FCs* and is supposed to significantly predict the actual use. However, it does not predict the BI (or acceptance), which is why it was not included in our regression model [33]. The results of this study supported the viability of UTAUT in determining the acceptance of e–mental health interventions. The three UTAUT predictors, *PE*, *EE*, and *SI*, achieved a total of 71.8% at the variance explanation of the acceptance of e–mental health interventions, and this is comparable with those of the original UTAUT validation study (70%) [33]. This study found that *PE* was the key predictor of acceptance [21,33,54-56], which is consistent with previous research suggesting that *PE* is also a predictor of treatment outcome in psychotherapy [57]. The implications of the strong relationship between *PE* and acceptance of e–mental health interventions are that there must be transparent eHealth education in which misunderstandings or false expectations are openly addressed.

Beyond the 3 UTAUT predictors, previous research has identified the following factors as predictors of e–mental health acceptance: *perceived reliability* [55], *stress owing to permanent availability* [21], *perceived security* [48], *technology anxiety* [54], and *resistance to change* [54]. The overall model in this study (with 16 predictors) was significantly better than the restrictive UTAUT model, but ultimately explained only slightly more variance in acceptance. In this study, complementing previous research on this topic, we could also find the *sum score of the psychometric instrument PHQ-8* (indicator for symptoms of depression), the *confidence in using digital media* as predictors of acceptance, and the *perceived stress through permanent availability.* In contrast to previous research, the regression coefficient of *perceived stress through permanent availability* in the overall model has a positive sign, which means that greater the stress perceived owing to constant accessibility, higher the acceptance was of e–mental health interventions. We explain this result as follows: people who report a high level of stress owing to permanent availability through their mobile phone use it very frequently, appreciate it, and use it for many different things in their everyday life. We assume that people who report a high level of stress from their mobile phone are particularly familiar with the functions and possibilities of their smartphone owing to the daily use. Therefore, they presumably exhibit a low inhibition threshold in the use of additional apps via smartphone and are more willing to use such technology for newly developed interventions or apps. People who reported low levels of stress from being available on their smartphone in the survey would also be unlikely to use their phone for e–mental health interventions because the smartphone does not have a significant role in their lives. This is a discovered difference from previous research, as, for example, Donkin and Glozier [58] identified technology fatigue as an important barrier to acceptance of e–mental health interventions and other findings, which also highlight that digital communication load is associated with psychological disorders such as burnout, anxiety, and depression [59]. *Confidence in using digital media* can be an influential variable concerning the acceptance of e–mental health interventions, as already shown by several studies related to internet use and internet literacy [60,61]. Again, the sign of the regression coefficient of *confidence in using digital media* is unexpected (negative), which, in interpretation first means that the more confident people are in using digital media, the less accepting they are of e–mental health interventions. However, when considering the entire regression model, it is noticeable that the sign of the regression coefficient for the predictor changes, which means that suppression effects might have occurred in the last step owing to the inclusion of the UTAUT factors. A practical implication resulting from this is to make as many patients as possible from different vulnerable patient groups familiar with the use of digital media and eHealth interventions so that they will be more widely accepted and used in the future.

Another new and important finding of this study is that psychometrics also contributes significantly to the variance explanation of acceptance of eHealth interventions among patients with obesity. Therefore, as a theoretical implication, this result should be included in the analysis of future research. The depression score (PHQ-8) is a significant predictor of the acceptance of e–mental health interventions in the overall regression model. As discussed above regarding mental disorders, we can assume that people with high depression scores and more prominent psychological symptoms generally experience more distress. This can lead to the finding that the acceptance of e–mental health interventions seems to be high among patients who are currently experiencing psychological distress.

The evaluation of the psychometric data (EDI-2–B, EDE-Q8, and PHQ-8) shows that the current sample of patients with obesity is noticeably psychologically burdened. The two kinds of psychological symptoms are eating disorders, such as binge eating, and depressive symptoms. A large review of obesity and psychiatric disorders from 2017 shows a very strong association between obesity and depression, especially in longitudinal studies, where the correlation was stronger for women. In addition, multiple studies have also shown an association between eating disorder symptoms and obesity [8]. This leads to the practical implication that eHealth interventions should be particularly targeted at restoring mental health, especially in patients with obesity.

In general, this study confirms recent findings (owing to high variance explanation of the 3 UTAUT factors in the acceptance of e–mental health interventions) and supplements them with further predictors (*depression, stress caused by permanent availability,* and *confidence in using digital media).*

However, it can be assumed that further factors influence the results of acceptance, especially the use of e–mental health interventions, and these must be taken into account when looking retrospectively at the results of previous research. In the evaluation of previous findings on acceptance and use of e–mental health interventions, we should consider whether the researchers used the UTAUT model or another measurement instrument to describe acceptance, the type and duration of the patient’s illness, the type of eHealth service, and whether patients or health care workers were surveyed.

### Limitations

The following limitations should be considered when interpreting the results of this study. As our study was exclusively web-based, it was mandatory that participants had internet access. As the spread of internet access varies, particularly between age groups, our sample of patients with obesity tends to be younger than the average in the general population (only 5 people older than 65 years). Moreover, the sex distribution is not representative of the overall population. As we recruited participants widely through social media groups (obesity surgery–related groups, which are almost exclusively composed of female members), a large number of women participated (403/448, 89.9% women and 45/448, 10% men), which restricts the generalizability of the results. In addition, we had varying numbers of patients in each obesity grade group: obesity grade I (82/448, 18.3%), obesity grade II (88/448, 19.6%), and obesity grade III (278/448, 62.1%). Thus, a significantly high number of persons are in obesity grade III, which also does not correspond to the distribution in society [62]. The BMI bias in this study (with 278/448, 62.1% of the participants with a BMI >40 kg/m^2^) is particularly important, as eHealth interventions often have preventive functions and could be valuable for patients in obesity groups I or II. Owing to these sampling biases, the generalizability of our results may once again be reduced. In addition to the unbalanced distribution of participants from the different obesity groups, we recruited a very high number of participants who had already undergone bariatric surgery (200/448, 44.6%) or were in the process of planning to undergo the surgery (170/448, 37.9%), which could also limit the generalizability of our results. Similarly, a considerable number of participants did not have a diagnosed mental health disorder at the time of the survey (which makes comparability difficult), whereby acceptance of e–mental health interventions was high among individuals with a mental health disorder. Nevertheless, the general psychological distress of the participants in this study was high, as measured by the valid psychometric instruments.

Owing to the recruitment of participants via the internet (in particular, via social media), we can assume that they might already have been more willing and interested in internet-related topics than random participants from different social backgrounds; therefore, we cannot rule out a selection bias. It is important to note that all the data collected were self-reported. Thus, accuracy of the results may be limited by the fact that participants may respond in a very socially elicited manner. Self-reporting can lead to a phenomenon known as common method bias [63]. To counteract this limitation, the instruments used in the study had sufficient reliability, the survey had a defensible length and was anonymous and web-based, and the patients were well educated, as these points are known to mitigate common method bias.

It is important to mention that this study, similar to most of the previous studies, determined the acceptance of e–mental health interventions among patients with obesity only by using the BI. A direct inference from the intention to use an e–mental health intervention to the actual use is not possible owing to the intention–behavior gap. However, further research should take the limitations of this study into account and include the actual use (behavior) of e–mental health interventions in patients with obesity and not focus only on acceptance.

As a theoretical implication for future research that also focuses on capturing the acceptance of e–mental health interventions, it would be particularly important to observe the distribution of sex, age, and the different obesity groups, to keep it as representative as possible. In addition, it would be beneficial to conduct a longitudinal study in which barriers and predictors for the actual use behavior of e–mental health services would be identified because no causality can be determined by cross-sectional studies. In addition, it would certainly be conceivable to survey other special patient groups, who, similar to patients with obesity (owing to stigma, physical illness, comorbidities, and immobility), have specific barriers that make the implementation of e–mental health interventions particularly important. This would facilitate the identification of the specific needs and demands of these patient groups and, as a practical implication, the development and implementation of e–health interventions that specifically target the improvement of mental and physical health.

### Conclusions

Although the measured acceptance in patients with obesity could be determined as moderate, this study highlights that the acceptance of e–mental health interventions differs significantly depending on the following variables: age, grade of obesity, occupational status, sex, and mental health status.

The UTAUT model with its three core predictors (*PE*, *EE*, and *SI*) has proven to be a valuable instrument to predict the acceptance of e–mental health interventions in patients with obesity. The variance explained by acceptance in the restrictive UTAUT model (the 3 core predictors) was high, but the extended UTAUT model is slightly but significantly better in comparison and highlighted three additional significant predictors (depression, stress owing to constant availability via mobile phone or email, and confidence in using digital media). Owing to the close association between acceptance and use, acceptance-facilitating interventions should be fostered to enhance the establishment of effective e–mental health interventions for patients with obesity. Low-threshold, location-flexible, and efficient e–mental health interventions are more important than ever before, especially with regard to the ongoing pandemic and in light of the high psychological vulnerability of patients with obesity. Especially because many patients with obesity decide to undergo bariatric surgery, such interventions could be very relevant in the preoperative phase (for psychological support) and in the postoperative follow-up. In addition, further research should be conducted to determine the detailed expectations, needs, and demands of patients with obesity regarding such tailor-made interventions to further increase motivation and acceptance.

## Appendix

Multimedia Appendix 1 Full hierarchical regression model of acceptance.

## Abbreviations

ANOVA: analysis of variance
BI: behavioral intention
EDE-Q8: Eating Disorder Examination-Questionnaire 8
EDI-2–B: Eating Disorder Inventory-2–Bulimia
EE: effort expectancy
FC: facilitating condition
PE: performance expectancy
PHQ-8: Patient Health Questionnaire-8
SI: social influence
UTAUT: Unified Theory of Acceptance and Use of Technology
