# Supplementary material for: Predicting Acceptance of e–Mental Health Interventions in Patients With Obesity by Using an Extended Unified Theory of Acceptance Model: Cross-sectional Study
Source: JMIR Form Res. 2022 Mar 17;6(3):e31229. doi: 10.2196/31229 (PMC8972105; doi:10.2196/31229)
Supplement: Multimedia Appendix 1 [file formative_v6i3e31229_app1.docx]

**Predicting Acceptance of e-Mental Health Interventions in Patients with Obesity by using an extended Unified Theory of Acceptance Model: Cross-sectional study**

Vanessa Rentrop^+1^, Mirjam Damerau^1^, Adam Schweda^1^, Jasmin Steinbach^1^, Lynik Chantal Schüren^2^, Marco Niedergethmann^2^, Eva-Maria Skoda^1^, Martin Teufel^1^, Alexander Bäuerle^1^

^1^University of Duisburg-Essen, Clinic for Psychosomatic Medicine and Psychotherapy, LVR-University Hospital Essen, 45147 Essen, Germany

^2^Department of General and Visceral Surgery, Alfried-Krupp Hospital Essen, 45131 Essen, Germany

^+^Corresponding author: Vanessa Rentrop, E-Mail: vanessa.rentrop@uni-due.de

**Multimedia Appendix 1**

**Table S1. Full hierarchical regression model of acceptance**

| **Predictor** | ***β*** | **B** | **T** | ***R*²** | **Δ *R*²** | ***P* value** |
| --- | --- | --- | --- | --- | --- | --- |
| Age | .035 | .004 | 1.339 | .736 | . 582 | .181 |
| Sex | -.013 | -.047 | - .494 |  |  | .622 |
| BMI | -.005 | -.001 | - .196 |  |  | .845 |
| Mental disorder  Occupational status | .006  -.046 | .007  -.110 | .212  -1.719 |  |  | .832  .086 |
| EDI-2-B | -.029 | -.005 | - .948 |  |  | .326 |
| PHQ-8 | .075 | .015 | 2.208 |  |  | .028 |
| EDE-Q8 | .029 | .026 | .935 |  |  | .350 |
| Stress due to permanent availability  Internet anxiety  Information about eHealth interventions  Experience with eHealth interventions  Confidence in using digital media | .063  -.043  .000  .048  -.058 | .057  -.063  .000  .135  -.067 | 2.273  -1.474  - .005  .1.741  -2.040 |  |  | .024  .141  .996  .082  .042 |
| Performance expectancy | .454 | .484 | 11.683 |  |  | <.001 |
| Effort expectancy | .219 | .268 | 5.913 |  |  | <.001 |
| Social influence | .271 | .361 | 7.956 |  |  | <.001 |
